# Topographic anatomy and morphometry of the gracilis muscle related to pelvic transposition flaps: Perineal versus transobturatory approach

**DOI:** 10.1016/j.jpra.2025.09.027

**Published:** 2025-09-25

**Authors:** Marvin Heimke, Tillmann Heinze, Julius Pochhammer, Roland Bertolini, Leonhard Buck, Thilo Wedel

**Affiliations:** a Institute of Anatomy, Center of Clinical Anatomy, Kiel University, Kiel, Germany; b Department of General, Visceral, Thoracic, Transplantation and Pediatric Surgery, University Hospital Schleswig-Holstein Campus Kiel, Kiel, Germany; c Department of Reconstructive Surgery, University Hospital Schleswig-Holstein Campus Kiel, Kiel, Germany; d Department of Urology, University Hospital Schleswig-Holstein Campus Kiel, Kiel, Germany; e Kurt-Semm Center for laparoscopic and robotic surgery, University Hospital Schleswig-Holstein Campus Kiel, Kiel, Germany

**Keywords:** Gracilis muscle flap, Pelvic reconstruction, Pelvic filling, Transobturatory transposition, Gracilis muscle vascular anatomy, Anatomical in-situ dissection

## Abstract

**Background:**

Pelvic dead space following major surgery is commonly addressed by vertical rectus abdominis muscle flaps, omental flaps, or gracilis muscle flaps. The drawback of the gracilis muscle flap is the limited amount of intrapelvic muscle tissue achieved by classic perineal transposition. This proof-of-principle study evaluates the transobturatory transposition of the gracilis muscle flap as an alternative to the perineal approach by revisiting the topographic and vascular anatomy and comparing the achievable intrapelvic muscle length.

**Methods:**

In-situ dissections were conducted in body donors (*n* = 38) and relevant morphometric data of the gracilis muscle were recorded. Perineal and transobturatory transpositions were compared for intrapelvic muscle length. Pedicle mobilization techniques (collateral vessel transection, adductor brevis muscle incision) were analyzed for their effectiveness in achieving maximal transposable muscle length.

**Results:**

The main vascular pedicle (mean length: 8.8 cm, mean entry point: 13.3 cm) of the gracilis muscle (mean length: 37.2 cm) originated predominantly from the deep femoral artery. Transobturatory transposition was technically feasible in all specimens, regardless of vascular pedicle pattern, and resulted in significantly increased mean intrapelvic muscle length compared to perineal transposition (17.8 vs. 14.5 cm). Further increase in intrapelvic muscle length was achieved by pedicle mobilization (20.9 vs. 15.5 cm) and incision of the adductor brevis muscle (additional gain of length: 1.0 cm).

**Conclusions:**

Gracilis muscle transposition via the obturator foramen is a viable alternative to the perineal route, achieving greater intrapelvic muscle length. The morphometric data on the topography and vasculature of the gracilis muscle provide a valuable basis to use the transobturatory transposition of the gracilis muscle for the reconstruction of pelvic defects.

## Introduction

Pelvic defects following major surgery for advanced anorectal malignancies, e.g., extralevator abdominoperineal rectal excision or pelvic exenteration, pose substantial reconstructive challenges.^1^ The remaining wound cavity delimited by bony pelvic boundaries predisposes to complications such as fluid accumulation, infection, wound dehiscence, fistula formation or bowel obstruction (“empty pelvis syndrome”),^1^^–^^3^ especially after preoperative irradiation.^4^ Increasing evidence suggests that pelvic filling by means of flap reconstruction may enhance primary wound healing and patient recovery.^3^^,^^5^, ^6^, ^7^

Commonly used flaps for pelvic reconstruction are the vertical rectus abdominis muscle flap (VRAM), the omental flap, and the gracilis muscle flap.^3^^,^^8^^,^^9^ Although the VRAM flap is commonly preferred due to its advantages (e.g., voluminous well-vascularized tissue mass, reliable vascular pedicle, wide arc of rotation),^10^ concerns regarding potential donor site morbidity have been raised.^11^ While minimally invasive surgical techniques have significantly reduced abdominal wall morbidity, employing the VRAM flap could still lead to increased complication rates such as abdominal wound rupture, fascial dehiscence and scar herniation.^1^^,^^9^^,^^12^^,^^13^ Furthermore, bilateral abdominal ostomies (e.g., enterostomy and urostomy) can make the VRAM flap less desirable.^14^^,^^15^ Additionally, severe abdominal adhesions from previous surgeries or infectious complications may necessitate a caudal approach for pelvic filling.^16^

The gracilis muscle flap represents an alternative with minimal donor site morbidity due to functional compensation by the remaining thigh adductors.^15^^,^^17^ The gracilis donor site typically lies outside the irradiation field and therefore provides healthy tissue for reconstruction without interfering with enterostomy procedures.^2^^,^^18^ However, a considerable limitation is the restricted amount of soft tissue, which may require bilateral harvesting to address larger volume deficits, particularly in the broader female pelvis.^15^

To enlarge the intrapelvic muscle mass, transposition of the gracilis flap via the obturator foramen may be considered as an alternative to the classic perineal approach. Although the transobturatory route is commonly used for suburethral tape implantation in female urinary incontinence treatment,^19^ this approach is only scarcely applied for gracilis muscle flaps and is not intuitive at first glance.^20^^,^^21^ Therefore, this anatomical proof-of-principle study revisits the topographic anatomy and vascular supply of the gracilis muscle by means of in-situ dissections and morphometric measurements, and evaluates the potential increase in intrapelvic muscle length achieved by transobturatory transposition compared to conventional perineal transposition.

## Materials and methods

### Body donors

Body donors were recruited from the local body donation program (Institute of Anatomy, Kiel University, Germany) after previous informed written consent to be used for medical research and educational purposes and approval by the local ethics committee (D 514/24, Medical Faculty, Kiel University). 38 lower extremities including the pelvic region were obtained from 19 body donors (10 females, nine males) with a mean age of 82 years (56–95 years), mean height of 164 cm (144–179 cm) and mean weight of 67 kg (37–117 kg). Previous diseases and surgical interventions involving the pelvis and lower extremities were excluded. Fixation was performed by perfusion of a solution containing formaldehyde (3 %), ethanol (75 %), and glycerine (7 %) via the femoral arteries and subsequent preservation by immersion in ethanol (70 %) prior to use.

### Macroscopic dissection and morphometric analysis

**Figure 1:**
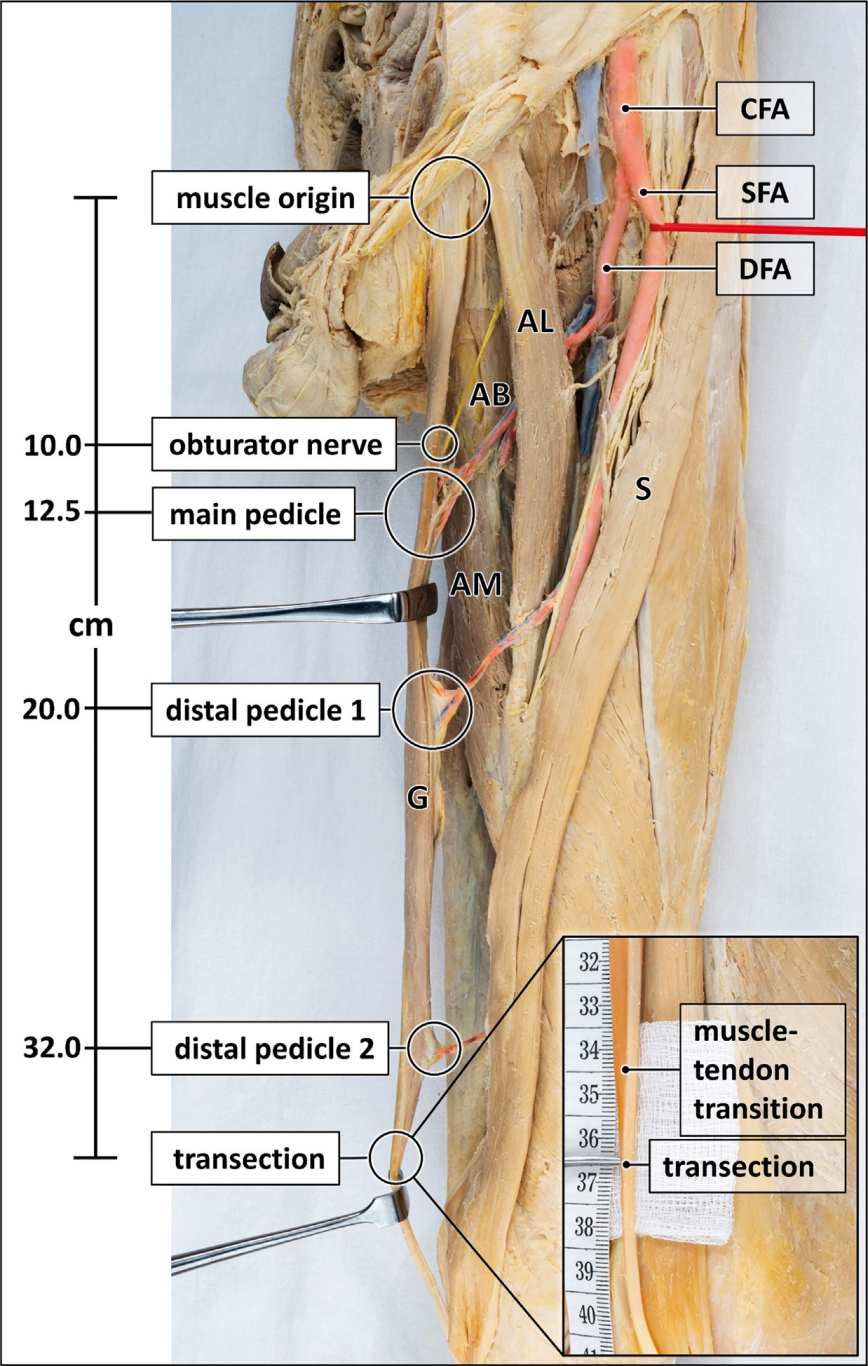
Measurement points for morphometric analysis. The gracilis muscle is distended with Langenbeck hooks to expose arteries (red), veins (blue) and nerve (yellow). Muscle length, length and entry points of the vascular and nervous supply are indicated. Some veins are removed for clarity. Inset magnifies the muscle-tendon transition and the transection point. AB = adductor brevis muscle; AL = adductor longus muscle; AM = adductor magnus muscle; CFA = common femoral artery; DFA = deep femoral artery; G = gracilis muscle; S = sartorius muscle; SFA = superficial femoral artery. Formalin-fixed left thigh and hemipelvis.

After removal of the skin and subcutis, the muscles, blood vessels and nerves of the thigh and pelvic region were exposed. The gracilis muscle along with its supplying blood vessels and the obturator nerve were meticulously dissected for in-situ morphometry of the following parameters (Figure 1). For selected parameters, variations in sample size occurred due to preliminary dissections or inadvertent injury resulting in loss of vascular or neural integrity.

#### Muscle length

The origin of the gracilis muscle was defined by its pubic insertion located immediately medial to the adductor longus tendon. The distal musculotendinous junction adjacent to the medial aspect of the knee joint was defined as the point where the composition was 50 % muscle/50 % tendon. For determining the muscle length used for the transposition flaps, the distal transection point was chosen 2 cm distal to the musculotendinous junction to provide a tendinous portion for anchoring sutures. Muscle length measurements were obtained in all specimens (*n* = 38).

#### Innervation

The origin, course and entry point of obturator nerve branches supplying the gracilis muscle were recorded (*n* = 33).

#### Main vascular pedicle

The origin, length, width, configuration, entry point into gracilis muscle, and collateral vessels to adductor muscles were recorded for the main vascular pedicle (*n* = 36). The most distal portion of the main pedicle was defined as the entry point of blood vessels into the muscle, as the surgical elevation of the gracilis muscle typically proceeds in distal to proximal direction.

#### Distal vascular pedicles

The presence, number, origin, and entry point of distal vascular pedicles additionally supplying the gracilis muscle were recorded (*n* = 32).

#### Accessory proximal vascular pedicle

If an accessory proximal vascular pedicle was present, its origin and entry point into the gracilis muscle were recorded (*n* = 38).

### Perineal and transobturatory transposition of the gracilis muscle

#### Perineal transposition flap

In 19 specimens the anorectum was resected from the pelvic cavity following the surgical principles and planes of total mesorectal excision (TME) applied for rectal carcinoma.^22^ Subsequently, the gracilis muscle was transected 2 cm distal to its musculotendinous junction and elevated in distal to proximal direction. All distal vascular pedicles were cut, while maintaining the main vascular pedicle. The perineal transposition into the pelvic cavity was carried out along the perineal body and followed the opening of the created pelvic defect (Figure 6, Figure 7).

#### Transobturatory transposition flap

After perineal transposition the same specimens were used for transobturatory transposition of the gracilis muscle. To reach the obturator foramen the adductor brevis muscle was retracted anterolaterally thereby exposing the external obturator muscle (Figure 8a). The soft tissues (external and internal obturator muscles, obturator membrane) covering the obturator foramen were then perforated at its craniomedial edge and bluntly widened to transpose the gracilis flap into the pelvic cavity without injuring the obturator vessels and nerve (Figure 6, Figure 7). Complete transposition into the pelvic cavity was achieved by pulling the muscle flap through a slit-like opening of the levator ani muscle (Figure 8b).

### Comparison of intrapelvic muscle length

The sagittal pelvic outlet diameter (line between the inferior border of the pubic symphysis and coccyx) served as a caudal reference point. The intrapelvic portion of the gracilis flap was pushed cranially crossing the sacrum between vertebra S1 and S2 to illustrate and measure the intrapelvic muscle length after perineal and transobturatory approach (*n* = 14).

To maximize intrapelvic muscle length, two additional procedures were performed: (1) The main vascular pedicle was completely mobilized by transecting collateral blood vessels which diverged into adjacent adductor muscles (*n* = 19) (Figure 9a). (2) The medial border of the adductor brevis muscle was incised to allow a tension-free shift of the main pedicle into cranial direction (*n* = 17) (Figure 9b).

### Photodocumentation and statistical analysis

Photographs were taken from all dissection steps (Sony Alpha 7.III, Sony FE 90 mm F2.8, Japan), and relevant structures were highlighted by superimposed semi-transparent colors (Adobe Photoshop Ver. 24.0.1, California). Morphometric data were analyzed with SPSS v29 (SPSS Inc., IBM Company, Armonk, NY, USA). The Shapiro-Wilk test was applied to test for normal distribution. Statistical comparison of intrapelvic muscle length between perineal and transobturatory transposition was performed by using the Wilcoxon-test, and gender differences were analyzed by using an independent *t*-test. The level of significance was set at 5 %. Results are expressed as mean, minimum and maximum values.

## Results

### Morphometric data

**Figure 2:**
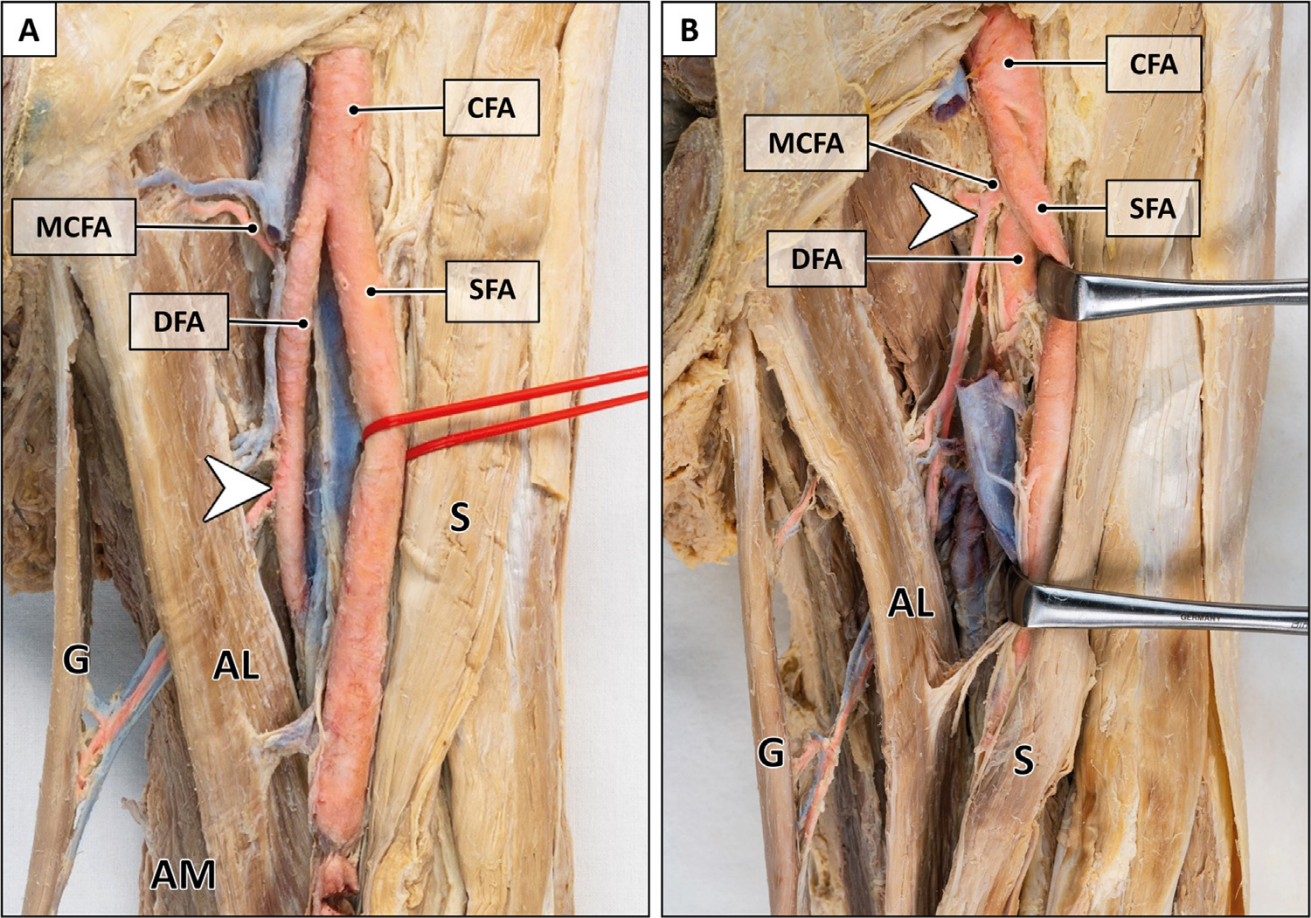
Origin of main pedicle. A: Origin of the gracilis artery from the deep femoral artery (arrowhead). B: Origin of the gracilis artery from the medial circumflex artery (arrowhead). AL = adductor longus muscle; AM = adductor magnus muscle; CFA = common femoral artery; DFA = deep femoral artery; G = gracilis muscle; MCFA = medial circumflex femoral artery; S = sartorius muscle; SFA = superficial femoral artery. Formalin-fixed left thigh.

**Figure 3:**
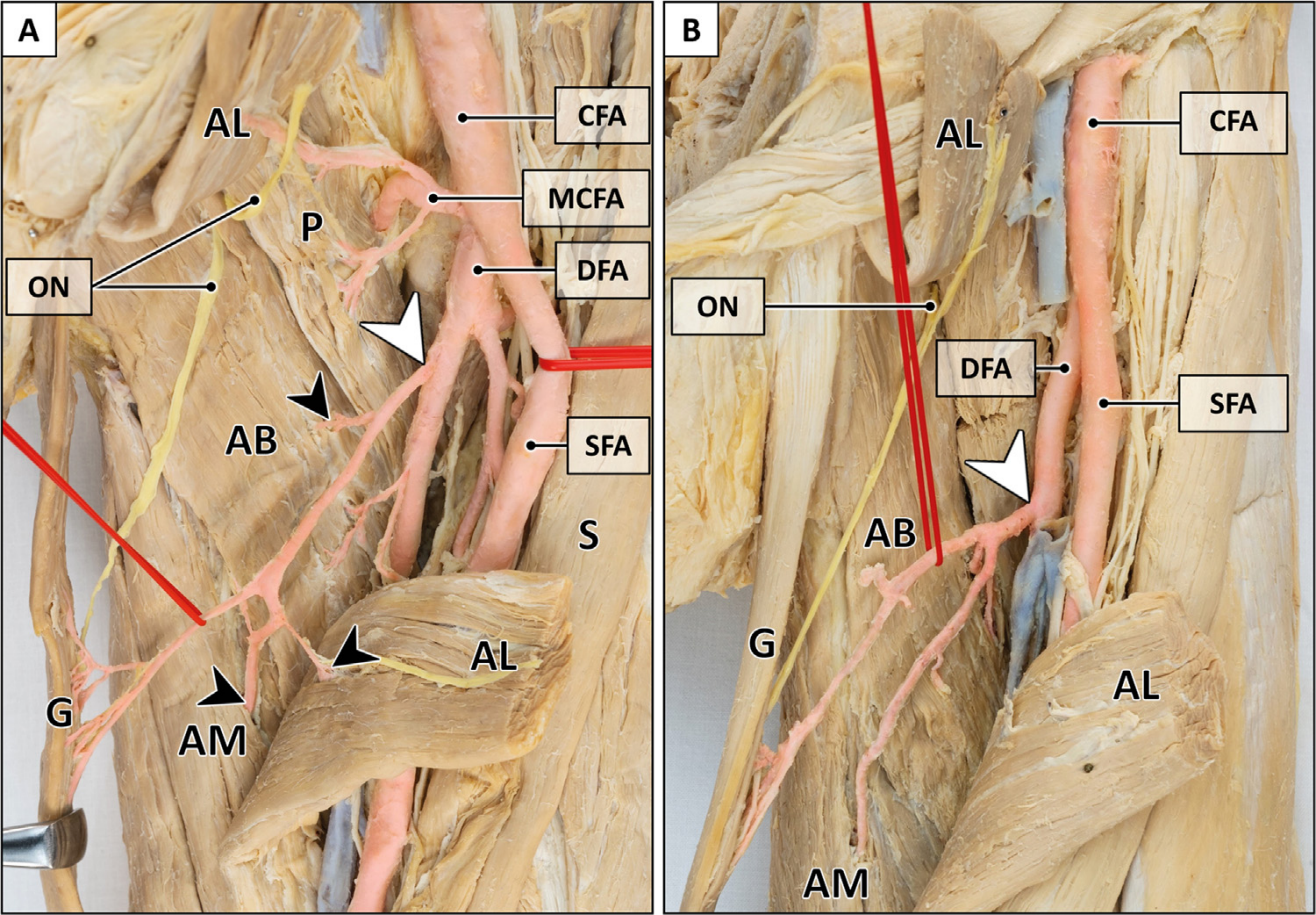
Variants of main pedicle. A: Solitary gracilis artery (white arrowhead) with collateral vessels (black arrowheads) to adductor muscles. B: Trunk-like pedicle (white arrowhead) splitting into the gracilis artery and arteries to adductor muscles. The adductor longus muscle is transected for better visualization of the main pedicle. AB = adductor brevis muscle; AM = adductor magnus muscle; AL = adductor longus muscle; CFA = common femoral artery; DFA = deep femoral artery; G = gracilis muscle; MCFA = medial circumflex femoral artery; ON = obturator nerve; P = pectineus muscle; S = sartorius muscle; SFA = superficial femoral artery. Formalin-fixed left thigh.

**Figure 4:**
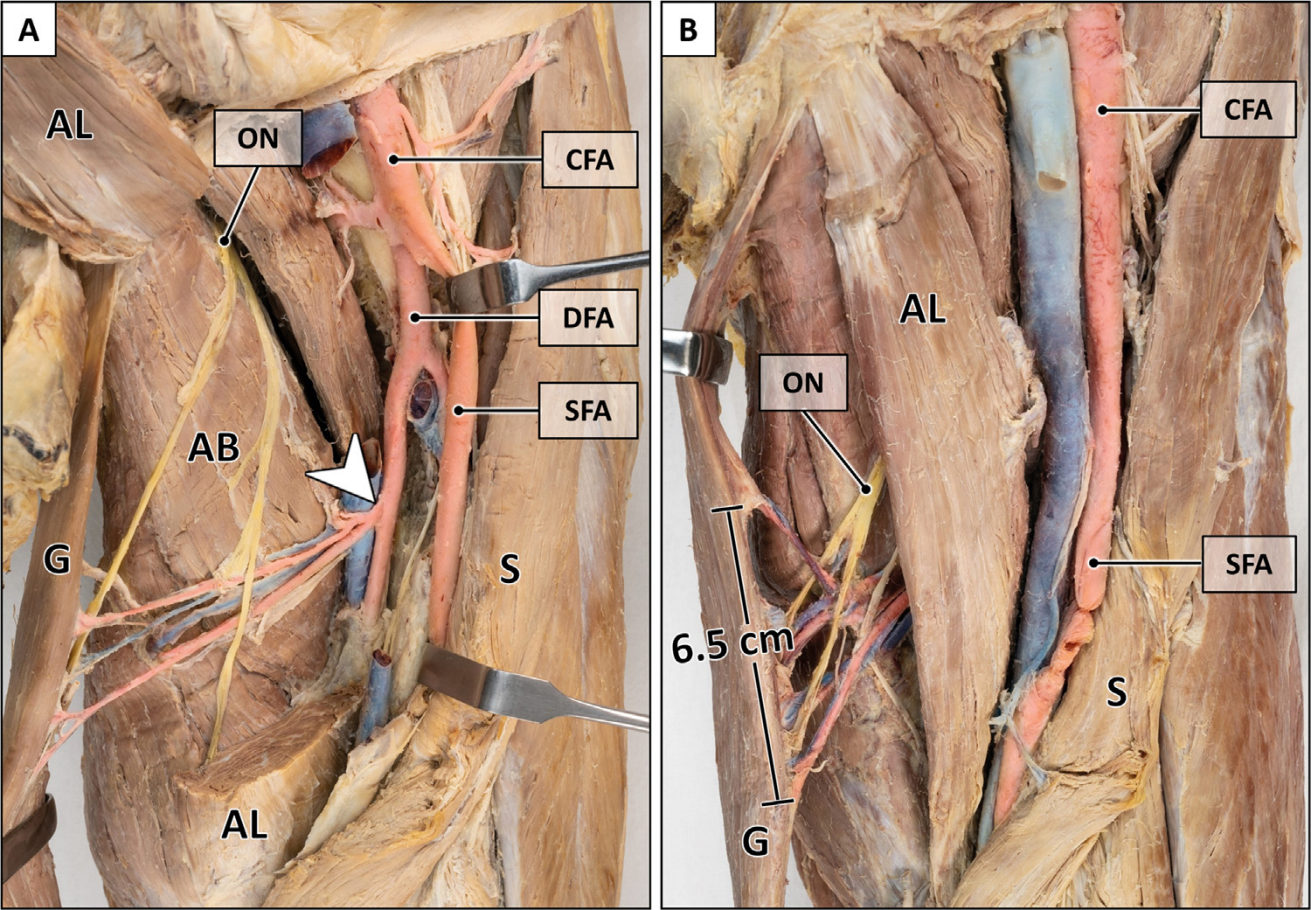
Peculiarities of main pedicle. A: Two main pedicles originating from a common trunk (arrowhead). The adductor longus muscle is transected for better visualization of the main pedicle. B: Broad entry site of the main pedicle (6.5 cm). AB = adductor brevis muscle; AL = adductor longus muscle; CFA = common femoral artery; DFA = deep femoral artery; G = gracilis muscle; ON = obturator nerve; S = sartorius muscle; SFA = superficial femoral artery. Formalin-fixed left thigh.

**Figure 5:**
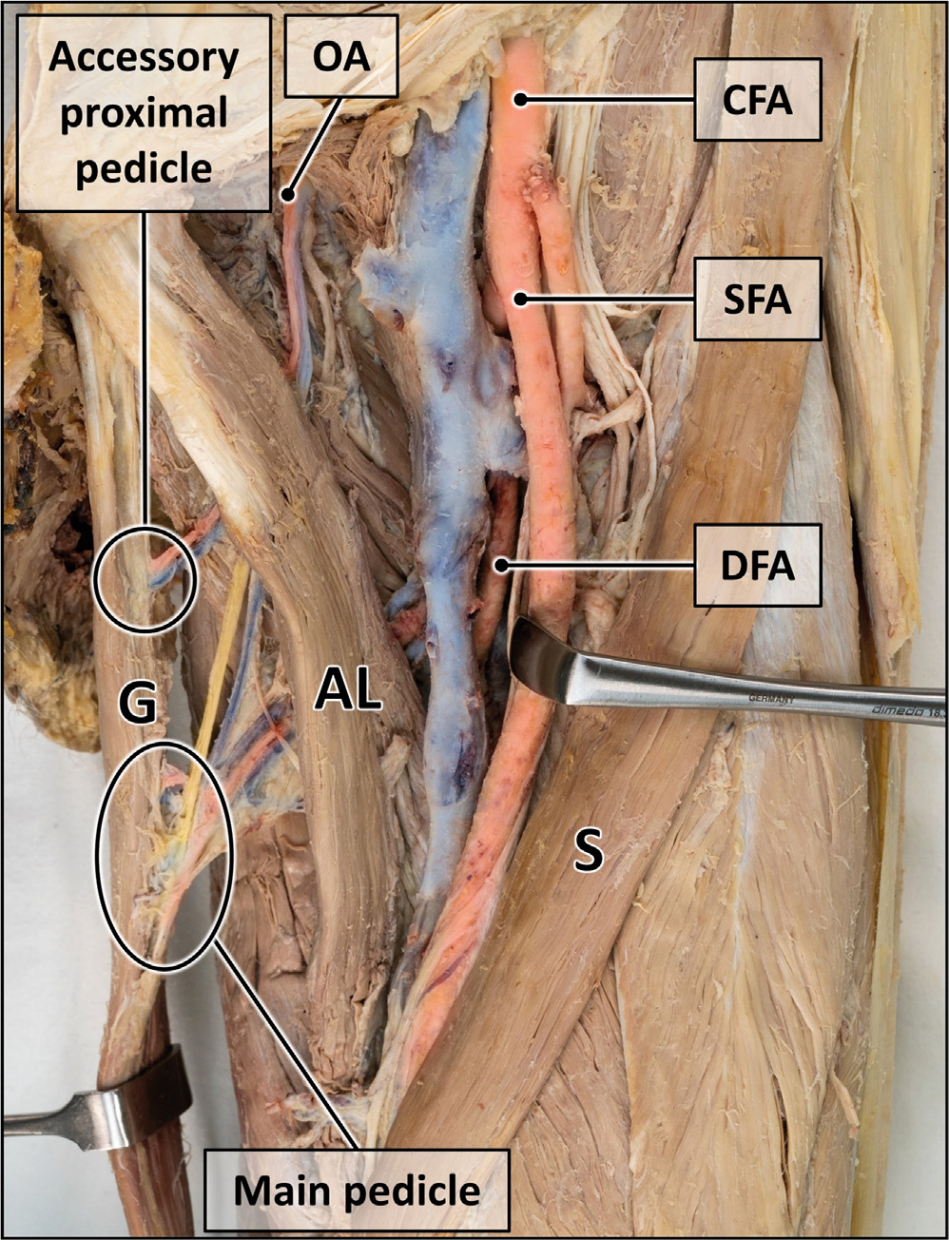
Proximal accessory pedicle. Accessory blood vessels originating from the obturator artery enter the gracilis muscle proximal to its main pedicle. AL = adductor longus muscle; CFA = common femoral artery; DFA = deep femoral artery; G = gracilis muscle; OA = obturator artery; S = sartorius muscle; SFA = superficial femoral artery. Formalin-fixed left thigh.

All morphometric data are displayed in Supplementary Table 1 and illustrated in Figure 1, Figure 2, Figure 3, Figure 4, Figure 5.

**Figure 6:**
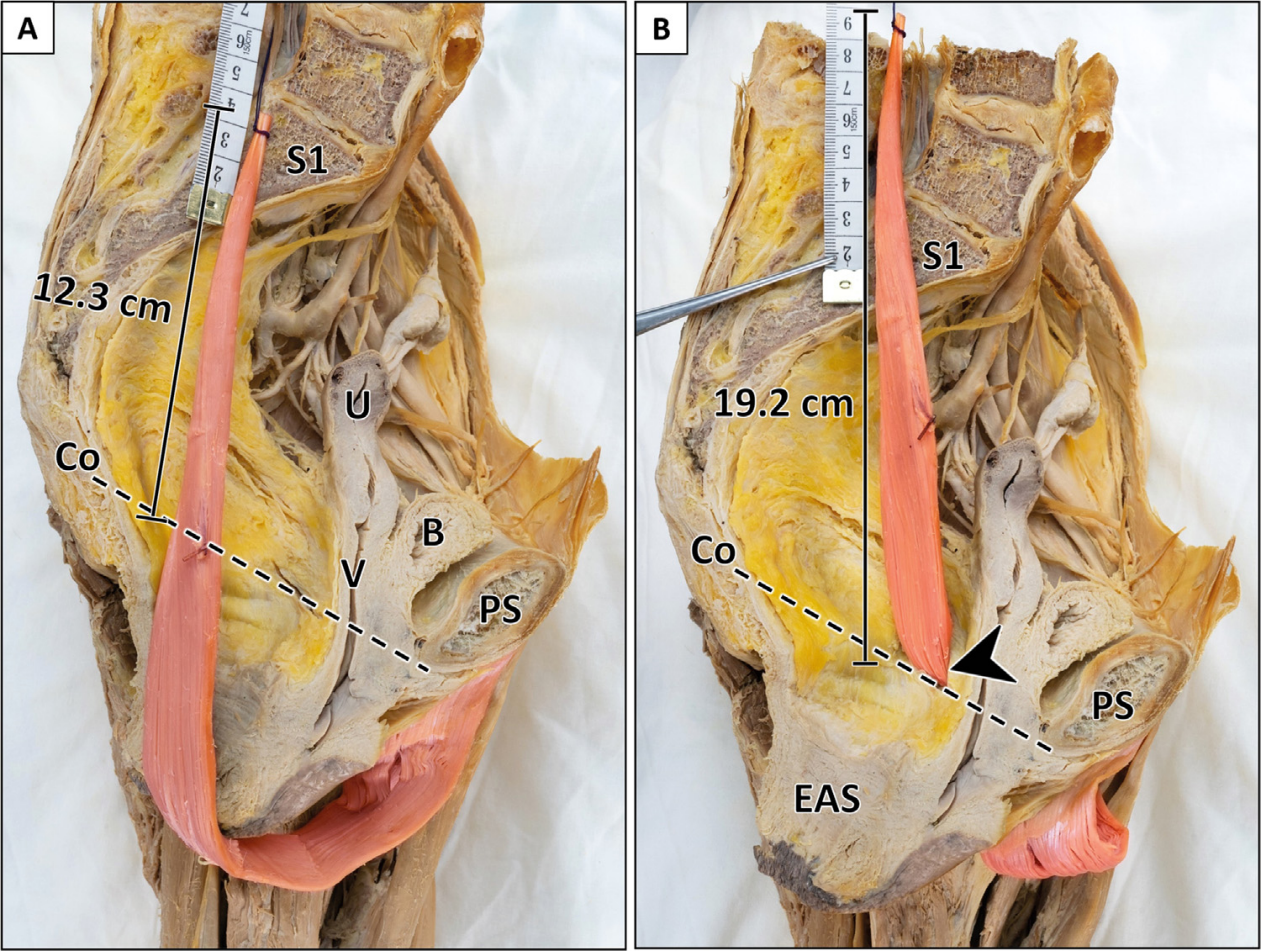
Comparison of intrapelvic muscle length after perineal and transobturatory transposition (female specimen). A: Perineal transposition of gracilis muscle yields an intrapelvic muscle length of 12.3 cm. B: Transobturatory transposition results in an intrapelvic muscle length of 19.2 cm with a gain in length of 6.9 cm. Arrowhead indicates transobturatory perforation site. The sagittal pelvic outlet diameter and the junction of sacral vertebra 1/2 served as reference points. B = bladder; Co = coccyx; EAS = external anal sphincter; PS = pubic symphysis; S1 = first sacral vertebra; U = uterus; V = vagina. Formalin-fixed left female hemipelvis after removal of the anorectum.

**Figure 7:**
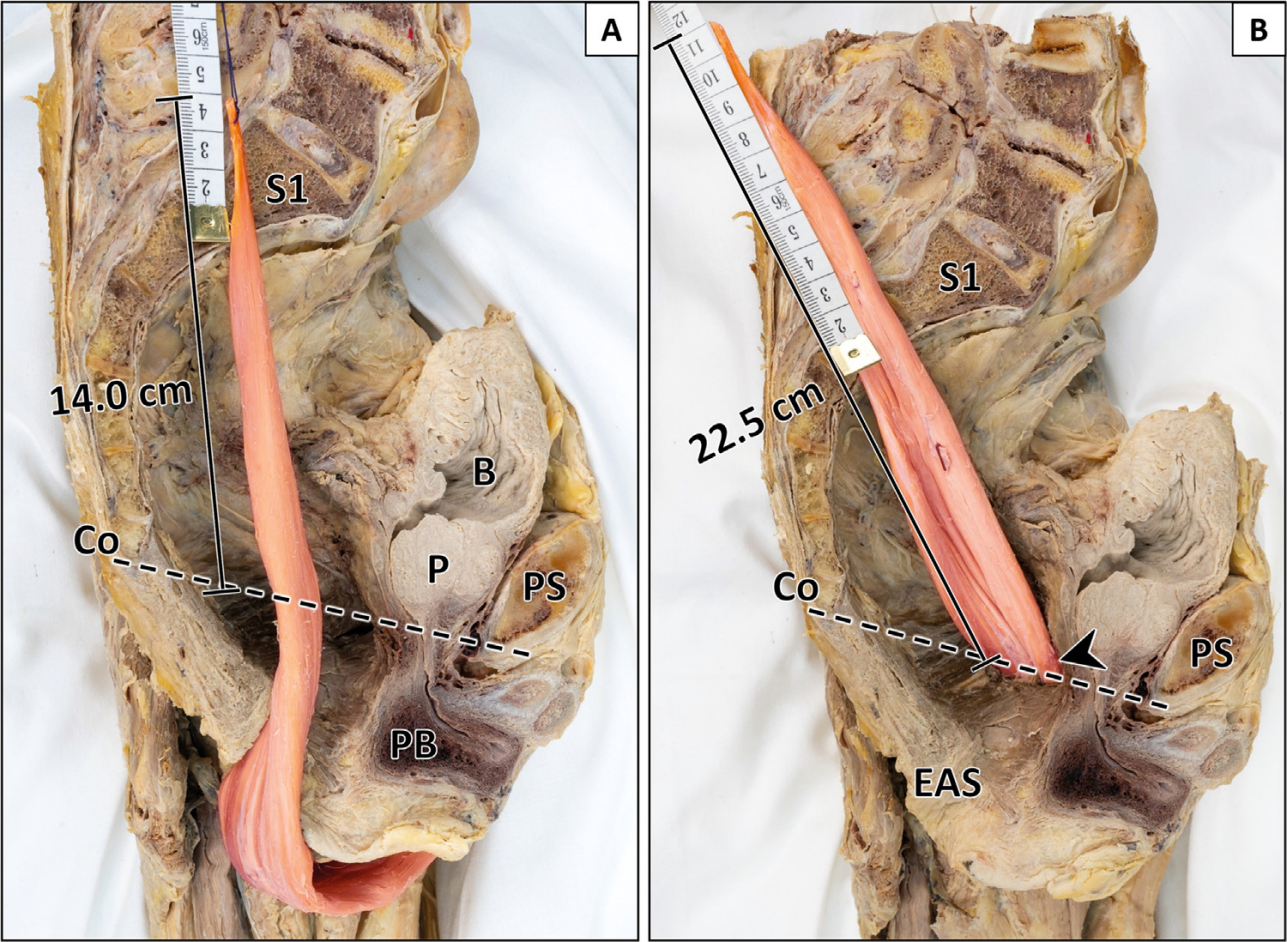
Comparison of intrapelvic muscle length after perineal and transobturatory transposition (male specimen). A: Perineal transposition of gracilis muscle yields an intrapelvic muscle length of 14.0 cm. B: Transobturatory transposition results in an intrapelvic muscle length of 22.5 cm with a gain in length of 8.5 cm. Arrowhead indicates transobturatory perforation site. The sagittal pelvic outlet diameter and the junction of sacral vertebra 1/2 served as reference points. B = bladder; Co = coccyx; EAS = external anal sphincter; P = prostate; PB = penile bulb; PS = pubic symphysis; S1 = first sacral vertebra. Formalin-fixed left male hemipelvis after removal of the anorectum.

**Figure 8:**
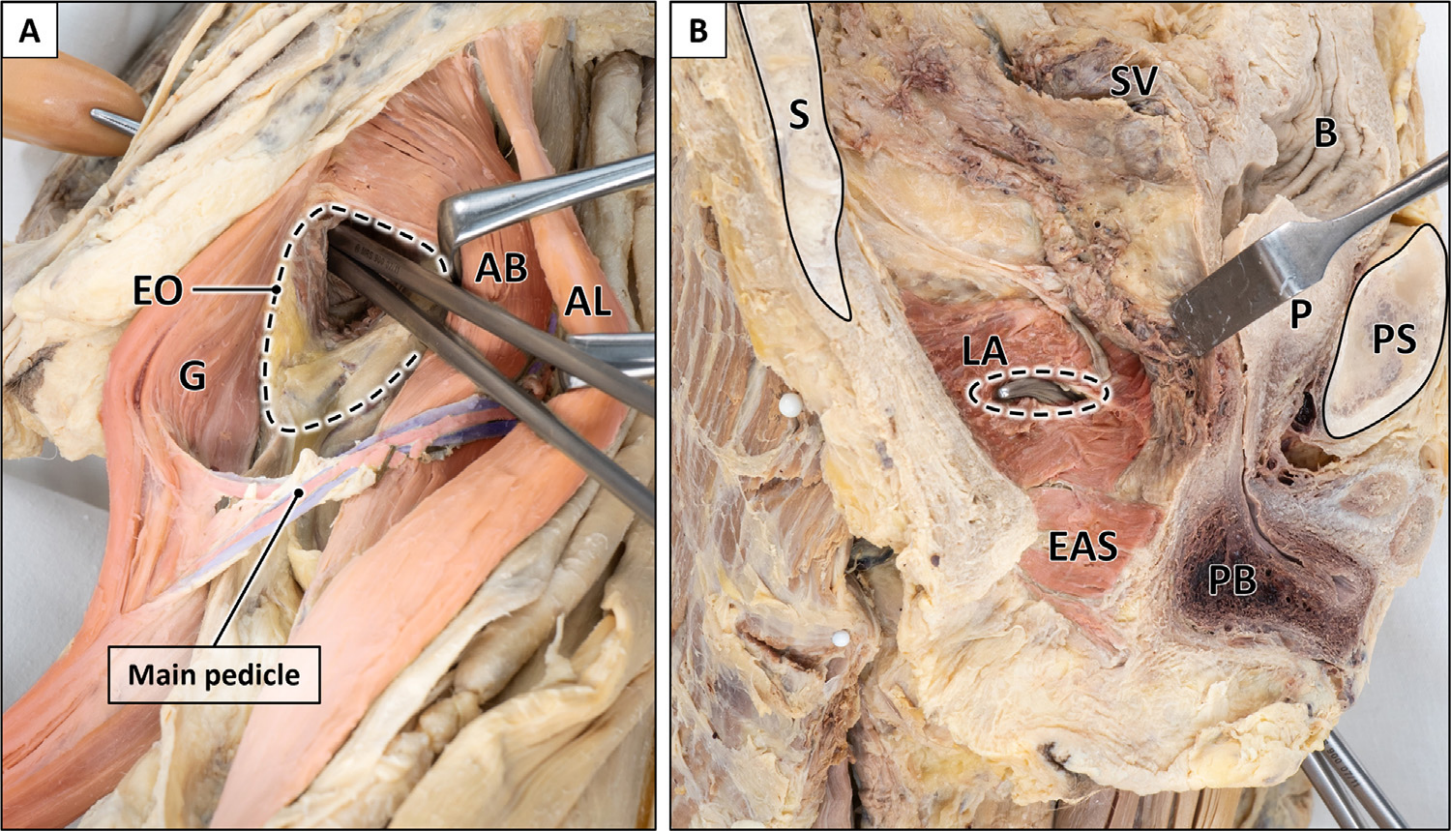
Transobturatory transposition route. A: Outside view (inner thigh region) after exposure of the external obturator muscle (dotted line) by retraction of the adductor brevis muscle. An Overholt forceps is passed through the obturator foramen medially to the adductor brevis muscle. B: Inside view (intrapelvic region) after piercing the levator ani muscle by an Overholt forceps (dotted line). AB = adductor brevis muscle; AL = adductor longus muscle; B = bladder; G = gracilis muscle; LA = levator ani muscle; P = prostate; PB = penile bulb; PS = pubic symphysis; S = sacrum; EAS = external anal sphincter; SV = seminal vesicle. Formalin-fixed left male hemipelvis with proximal thigh.

#### Muscle length

The length of the gracilis muscle used for the flap, measured from its origin to the transection point (2 cm distal from the musculotendinous junction), was 37.2 cm (34.0–40.0 cm).

#### Main pedicle

The main pedicle originated either from the deep femoral artery (94.4 %), the superficial femoral artery (2.8 %), or the medial circumflex artery (2.8 %) (Figure 2). Variations of the primary pedicle arising from the deep femoral artery comprised a prominent artery running towards the gracilis muscle with small collateral branches for other adductors (Figure 3a), or a trunk-like pedicle which divided into evenly sized branches supplying the gracilis and adjacent adductor muscles (Figure 3b).

The main pedicle approached the muscle craniolaterally running between the adductor longus and adductor brevis muscle and entered into the gracilis muscle at 13.3 cm (10.0–17.0 cm) from its pubic origin. Its width at the entry point ranged from 0.5 to 6.5 cm (mean: 2.5 cm) (Figure 4b). The average length of the main pedicle was 8.8 cm (5.1–14.6 cm). Collateral branches consistently supplied the adductor longus (97.4 %), adductor magnus (21.1 %), and adductor brevis muscle (13.2 %) (Figure 3a).

#### Distal pedicles

Blood vessels entering into the gracilis muscle distal to the main pedicle (Figure 1) were identified in all specimens. One distal pedicle was present in 34.4 %, two in 46.8 %, three in 12.5 %, and four in 6.3 % of cases.

#### Proximal accessory pedicle

A small-caliber vascular pedicle proximal to the main pedicle was observed in 28.9 % of specimens (Figure 5), entering the gracilis muscle at 6.6 cm (3.5–12.5 cm). The pedicle approached the muscle craniolaterally and originated mainly from the obturator artery (77.8 %) and less frequently from the deep femoral artery (11.1 %) or medial circumflex artery (11.1 %).

#### Innervation

In all specimens, the gracilis muscle was innervated by the anterior branch of the obturator nerve (Figure 4a). The nerve passed anterior to the adductor brevis muscle and entered into the gracilis muscle at 11.2 cm (9.0–14.0 cm), at the proximal portion of the main vascular pedicle.

### Perineal versus transobturatory transposition

#### Intrapelvic muscle length without pedicle mobilization

First, perineal and transobturatory transposition of the gracilis muscle were performed without mobilization of the main pedicle. The intrapelvic muscle length following perineal transposition yielded 14.5 cm (9.5–18.0 cm). After transobturatory transposition, the intrapelvic muscle length significantly increased to 17.8 cm (14.0–22.5 cm) (Supplementary Table 2). Both approaches were feasible in all specimens regardless of the vascular anatomy.

#### Intrapelvic muscle length with pedicle mobilization

**Figure 9:**
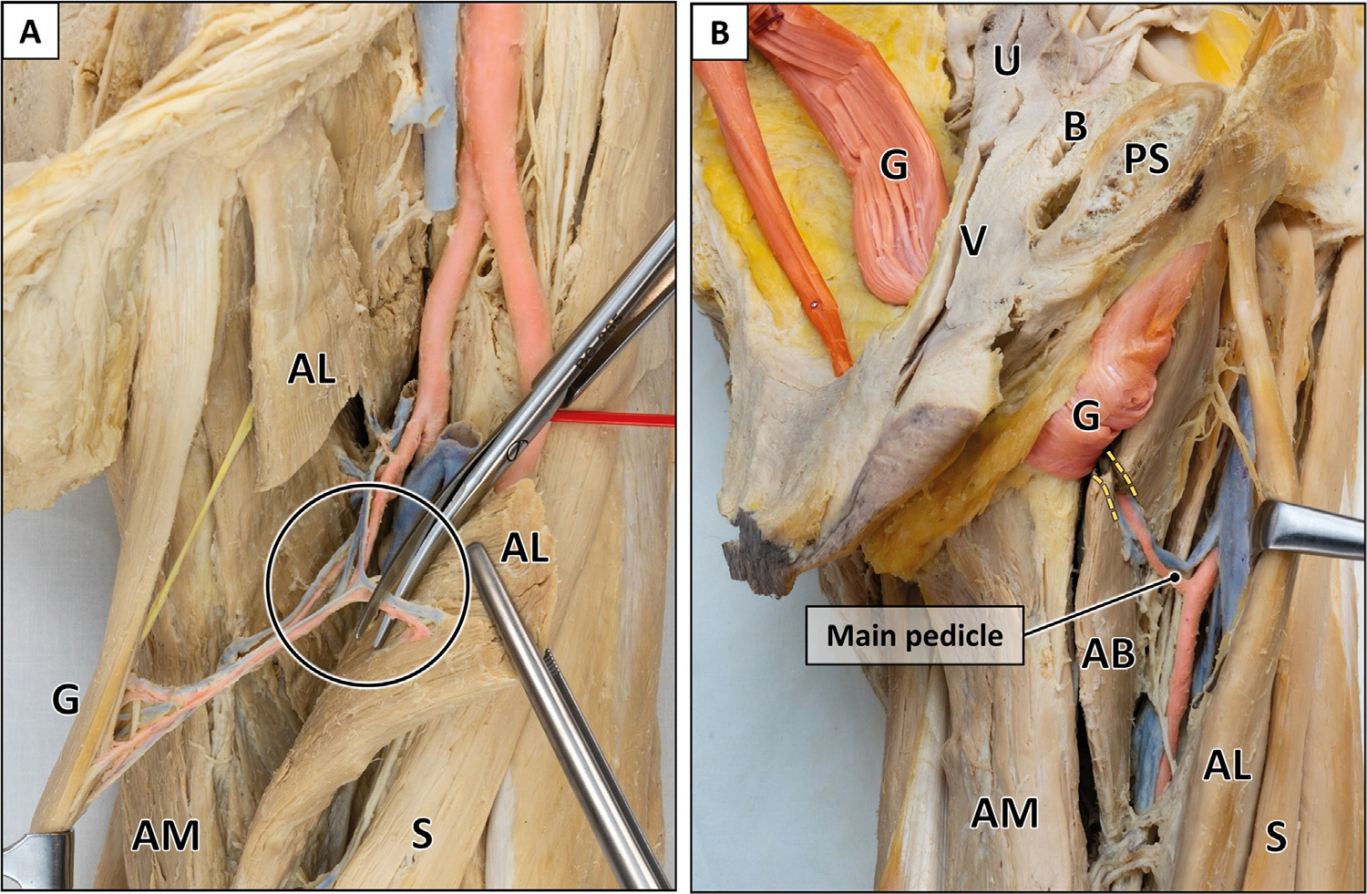
Techniques for additional gain of muscle length. A: Mobilization of the main pedicle by transection of collateral vessels originating from the gracilis artery. The adductor longus muscle is transected for better visualization of the main pedicle. B: Incision of the adductor brevis muscle (yellow dotted lines) to further facilitate pedicle mobilization in cranial direction. AB = adductor brevis muscle; AL = adductor longus muscle; AM = adductor magnus muscle; B = bladder; G = gracilis muscle; PS = pubic symphysis; U = uterus; V = vagina; S = sartorius muscle. Formalin-fixed left thighs.

Second, the main pedicle was mobilized by transecting its collateral vessels (Figure 9a). This procedure resulted in an additional gain of intrapelvic muscle length for both approaches, but was significantly larger following transobturatory transposition (2.8 cm) compared to perineal transposition (1.2 cm). Accordingly, total intrapelvic muscle length after pedicle mobilization increased to 15.5 cm (9.5–19.8 cm) for perineal and 20.9 cm (14.0–25.3 cm) for transobturatory transposition (Supplementary Table 2). The greater intrapelvic muscle length achieved by the transobturatory approach was attributable to the main pedicle entering the pelvis in a rather straight trajectory, whereas in perineal transposition it followed a curved path around the adductor muscles and inferior pubic ramus, limiting further advancement.

A comparative case-related analysis after pedicle mobilization yielded an individual muscle length gain of 5.4 cm (1.0–9.4 cm) for the transobturatory approach compared to the perineal approach. Noteworthy, in 10.1 % of cases, a greater intrapelvic muscle length for the transobturatory approach was only achievable if the main pedicle was fully mobilized. Furthermore, incision of the adductor brevis muscle contributed to an additional average increase of 1.0 cm, allowing for a final intrapelvic flap length of 21.8 cm (16.0–26.0 cm) achieved by the transobturatory approach (Figure 9b). Comparable gain of muscle length was achieved in both male and female specimens, with no statistically significant difference.

## Discussion

The anatomical hallmarks of the gracilis muscle have been investigated by different techniques, including radiographic analysis,^23^, ^24^, ^25^, ^26^, ^27^, ^28^, ^29^, ^30^ ex-situ investigations,^24^^,^^26^, ^27^, ^28^, ^29^^,^^31^^,^^32^ perfusion studies,^24^^,^^26^, ^27^, ^28^, ^29^, ^30^^,^^32^, ^33^, ^34^ and macroscopic dissections performed in body donors.^25^^,^^28^, ^29^, ^30^^,^^33^, ^34^, ^35^, ^36^ In this study, we focused first on meticulous in-situ dissections to provide a detailed photodocumentation of the retrieved morphometric data, and then addressed the intrapelvic muscle length achieved by either perineal or transobturatory transposition of the gracilis muscle.

### Topographic anatomy and morphometric data

#### Muscle length

Previous studies reported a mean muscle length ranging from 27.9 to 46.9 cm.^24^^,^^25^^,^^29^^,^^30^^,^^34^^,^^35^ The large spread most likely results from differing definitions of origin and insertion. For the present clinical purpose, we defined the muscle origin directly medial to the palpable adductor longus tendon and the distal point 2 cm beyond the musculotendinous junction. Based on these reference points, the mean muscle length suitable for reconstructive flap surgery yielded 37.2 cm (34.0–40.0 cm).

#### Main pedicle

In general, our findings on the vascular supply of the gracilis muscle are consistent with the literature.^23^, ^24^, ^25^^,^^27^, ^28^, ^29^, ^30^, ^31^, ^32^, ^33^, ^34^, ^35^, ^36^ Whitaker et al. and Magden et al. reported the deep femoral artery as the primary origin of the main pedicle (87–93 %) and, less frequently, the medial circumflex artery (7–13 %).^23^^,^^33^ Similarly, we found the main pedicle originating from the deep femoral artery in 94 %, and from either the medial circumflex femoral artery or superficial femoral artery in 3 % of cases.

Some authors have described an 'artery of adductors'—a transverse branch from the deep femoral artery—as the origin of the main pedicle.^24^^,^^28^^,^^32^^,^^34^^,^^37^ In these studies, the main pedicle originated either from this artery of adductors (33–81 %) or directly from the deep femoral artery (7–54 %).^24^^,^^32^^,^^34^ Our dissections showed that the gracilis artery consistently provided collateral branches to other adductors: either as a dominant artery with small side branches (Figure 3a) or as a common trunk dividing into evenly sized branches (Figure 3b). This finding may explain the variability reported in the literature. Due to the difficulty to objectively distinguish between a solitary gracilis artery with collateral branches and a distinct 'artery of adductors', we did not differentiate further in this study.

The mean entry point of the main pedicle reported in the literature ranged from 8 to 16.3 cm, depending on the anatomical reference points defined as muscle origin.^23^, ^24^, ^25^^,^^27^, ^28^, ^29^, ^30^, ^31^^,^^33^, ^34^, ^35^ In our study, the mean distance between the origin of the gracilis muscle medial to the adductor longus tendon and the most distal portion of the pedicle was 13.3 cm (10.0–17.0 cm). Given the variable width of the pedicle (Figure 4b), the incorporation of the most distal entry point is crucial to prevent potential vascular injuries, as the gracilis muscle is typically elevated in distal to proximal direction. The mean pedicle length of 8.8 cm was slightly longer than the reported average lengths in previous studies (7.6–8.2 cm).^30^^,^^34^

#### Distal pedicles

The gracilis muscle is reported to be supplied by two,^29^^,^^33^ three,^23^^,^^26^^,^^27^^,^^30^, ^31^, ^32^ or four^24^ distal pedicles, originating from either the superficial femoral artery or, more distally, the popliteal artery. We could confirm the presence of one to four distal pedicles from these origin sites, with two distal pedicles being the most common finding (46.9 %).

#### Proximal accessory pedicle

A proximal accessory pedicle has been reported with a frequency ranging from 8 % to 100 %.^23^, ^24^, ^25^^,^^28^^,^^30^, ^31^, ^32^, ^33^, ^34^, ^35^ In our study, we identified a proximal accessory pedicle in 28.9 % with a mean entry point at 6.6 cm consistent with previously reported data (5.7–6.6 cm).^30^^,^^33^^,^^35^ Most commonly, we found the obturator artery as origin in accordance with prior reports.^23^^,^^25^^,^^30^^,^^32^^,^^35^^,^^36^ However, some studies have identified the medial circumflex artery,^27^^,^^33^ common femoral artery,^28^ or deep femoral artery^24^ as predominant origin.

### Comparison between perineal and transobturatory transposition

The gracilis muscle flap is widely utilized in reconstructive procedures, including the reconstruction of pelvic defects.^8^^,^^15^ A notable limitation of this flap is the restricted tissue volume, which may require bilateral harvesting to address larger volume deficits.^15^ Consequently, the VRAM flap is often preferred for pelvic filling following extensive surgeries such as extralevator abdominoperineal excision of the rectum or pelvic exenteration.^18^ However, the use of the VRAM flap becomes less appropriate, if the inferior epigastric vessels have been compromised by prior surgical interventions, abnormal scarring or a ventral hernia is present, bilateral abdominal wall ostomies are planned, or severe abdominal adhesions necessitate a caudal approach for reconstruction.^8^^,^^14^, ^15^, ^16^ Moreover, donor site morbidity following the VRAM flap includes the risk of scar hernia.^38^ Under these circumstances, and in cases of VRAM complications, the gracilis flap stands out as a preferable alternative, in particular due to its minimal donor site morbidity and the fact that the muscle remains outside the radiation field, thus offering healthy tissue conditions for reconstruction.^2^^,^^9^^,^^18^

**Figure 10:**
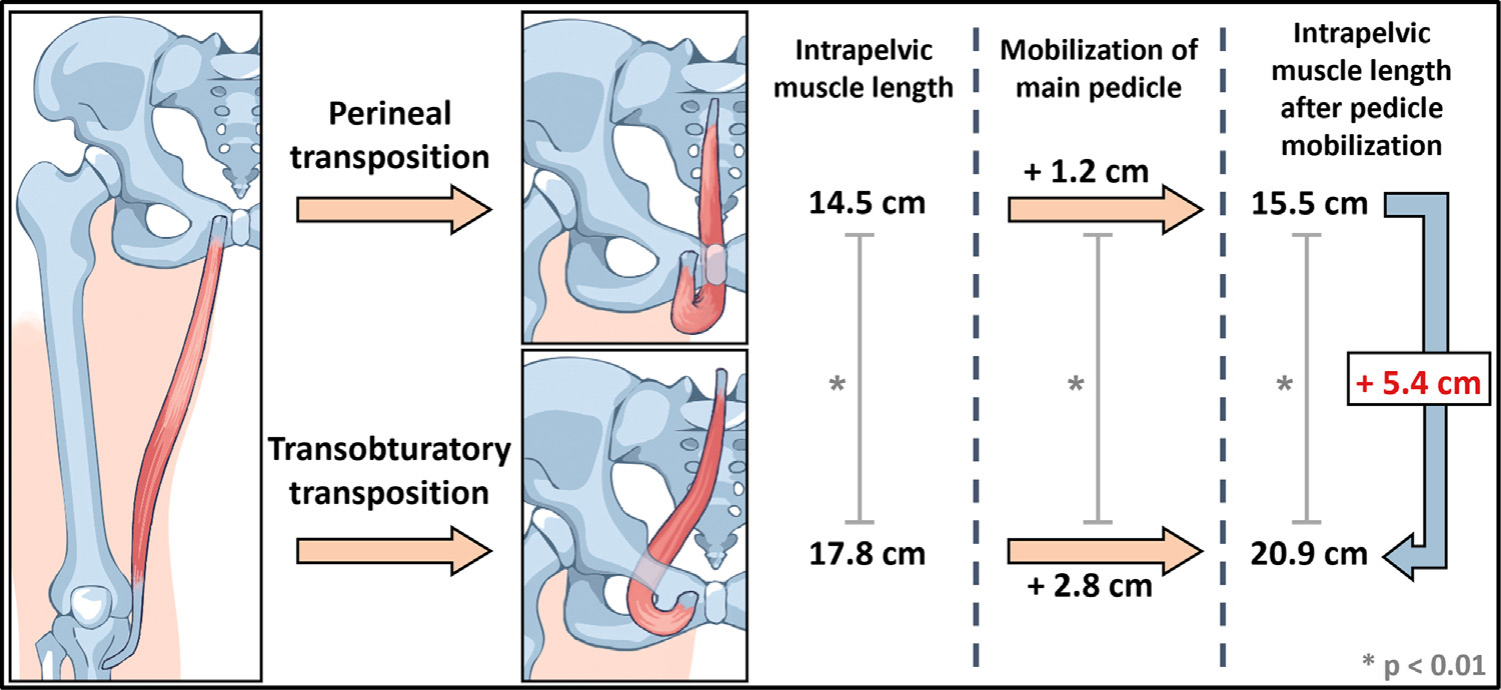
Intrapelvic muscle length gain by transobturatory vs. perineal transposition. Transobturatory transposition results in a significantly longer mean intrapelvic muscle length (17.8 cm) compared to the perineal approach (14.5 cm). Additional pedicle mobilization leads to a significantly greater mean muscle length gain in the transobturatory transposition (2.8 cm) compared to the perineal approach (1.2 cm). The total mean intrapelvic muscle length achieved after pedicle mobilization was 20.9 for the transobturatory transposition and 15.5 cm for the perineal transposition with a significant difference of 5.4 cm.

Conventionally, the gracilis muscle is transposed via the perineal route for pelvic defect reconstruction.^39^^,^^40^ In a previous single-center study, the gracilis muscle was transposed via a transobturatory approach for total anal reconstruction, positioning the muscle in a U-shape around the (neo)rectum to simulate the puborectalis muscle.^20^^,^^21^ Our findings confirm that the transobturatory approach is feasible for pelvic defect filling, irrespective of anatomical variations of the main vascular pedicle. Moreover, our study provides evidence that this approach yields significantly increased intrapelvic muscle length in both females and males by omitting the “perineal loop” (Figure 10). Noteworthy, as the muscle is inserted into the pelvis from its distal end, each centimeter of additionally gained intrapelvic length translates into a substantial increase in tissue volume, due to the muscle’s progressive proximal widening. However, to achieve maximal transposable muscle length, the main pedicle has to be completely mobilized and the adductor brevis muscle has to be incised medially. As the transobturatory route provides direct access of the gracilis muscle into the pelvic cavity, this approach is expected to allow a more effective filling of intrapelvic defects and an anatomically stable positioning of the muscle flap.

A limitation of this study is the use of formalin-fixed specimens. Reduced tissue elasticity may affect the realistic assessment of muscle dynamics and transpositional mobility.^41^ However, it is likely that the intrapelvic muscle length would be comparable or even greater in vivo, due to the higher flexibility of unfixed tissue. As both types of muscle transposition (perineal and transobturatory approach) were carried out in each specimen, the relative difference of resulting intrapelvic muscle length could be extrapolated to the in-vivo situation. Moreover, assessment of the perfusion quality following muscle transposition is not feasible in formalin-fixed specimens and requires clinical evaluation.

## Conclusion

In a first step, this study has revisited the topographic anatomy of the gracilis muscle by providing detailed morphometric data and a comprehensive photodocumentation of in-situ dissections. In a second step, these findings were used to verify that a transobturatory transposition of the gracilis muscle results in a significant gain of intrapelvic muscle length compared to the conventional perineal approach. The considerably increased intrapelvic muscle length suggests that an unilateral transobturatory gracilis muscle flap may be a valuable alternative for standard flap procedures to fill intrapelvic defects following major pelvic surgery. Whether the transobturatory transposition route of the gracilis muscle illustrated in this proof-of-principle setting is applicable in a clinical context, has to be addressed by further studies.

## Funding

This study received no funding.

## Ethical approval

The body donors were recruited from the local body donation program (Institute of Anatomy, Kiel University, Germany) after previous informed written consent to be used for medical research and educational purposes in accordance with the regulatory guidelines (Burial Act of the State of Schleswig-Holstein, Section II, § 9) and approval by the ethics committee (D 514/24, Medical Faculty, Kiel University).

## Data availability

Data available on request from the authors.

## Declaration of competing interest

The authors certify that they have no affiliations with or involvement in any organization or entity with any financial interest (such as honoraria; educational grants; participation in speakers’ bureaus; membership, employment, consultancies, stock ownership or other equity interest; and expert testimony or patent-licensing arrangements) or nonfinancial interest (such as personal or professional relationships, affiliations, knowledge or beliefs) in the subject matter or materials discussed in this manuscript.
